# Distant delivery of a mindfulness-based intervention for people with Parkinson’s disease: the study protocol of a randomised pilot trial

**DOI:** 10.1186/s40814-016-0117-4

**Published:** 2017-01-16

**Authors:** A. Bogosian, C. S. Hurt, D. Vasconcelos e Sa, J. V. Hindle, L. McCracken, P. Cubi-Molla

**Affiliations:** 1 Division of Health Services Research & Management, School of Health Sciences, City, University of London, EC1V 0HB London, UK; 2 School of Psychology, Bangor University, Bangor, UK; 3 Health Psychology, Institute of Psychiatry, Psychology & Neuroscience, King’s College London, London, UK; 4 Office of Health Economics, London, UK

**Keywords:** Randomised controlled trial, Parkinson’s disease, Anxiety, Depression, Mindfulness

## Abstract

**Background:**

Psychological difficulties, especially depression and anxiety, are the most prevalent non-motor symptoms in Parkinson’s disease. Pharmacological treatments for these conditions appear relatively ineffective in Parkinson’s disease. Mindfulness courses are increasingly popular and recognised as effective for managing emotional states, and there is growing evidence for the effectiveness of mindfulness courses for people with long-term medical conditions. With this exploratory pilot trial, we want to assess the feasibility of the procedures and processes, including recruitment, most appropriate outcome measure(s), acceptability of type and number of measures, potential nocebo effects, and potential effectiveness and cost-effectiveness of a specially adapted distance-delivered mindfulness-based intervention in people affected by Parkinson’s disease.

**Methods/Design:**

This is a pilot two-arm randomised parallel group controlled trial. Sixty participants who meet eligibility criteria will be randomly assigned either to an 8-week mindfulness-based intervention group or a wait-list control group. The mindfulness intervention will include 1-h weekly sessions delivered by a health psychologist trained to facilitate mindfulness courses. Participants in both groups will complete standardised questionnaires assessing anxiety, depression, pain, insomnia, fatigue, and daily activities at four time points (baseline, 4, 8, and 20 weeks). The analysis will also consider potential mechanisms of change, such as acceptance, self-compassion, and tolerance of uncertainty, as well as health economic outcomes. Participants’ experiences of the mindfulness interventions will be explored via in-depth interviews.

**Discussion:**

A mindfulness-based intervention for people with Parkinson’s delivered remotely, through Skype group videoconferences, may represent a viable, more accessible, intervention for people with mobility limitations and people who live in rural areas. The trial will provide important information about the feasibility, potential efficacy and cost-effectiveness, and acceptability of the intervention as well as mechanisms of psychosocial adjustment. The results of this pilot trial will help us design a phase III trial to assess efficacy of an online mindfulness-based intervention in Parkinson’s disease and evaluate significance.

**Trial registration:**

ClinicalTrials.gov, NCT02683330

## Background

The non-motor symptoms of Parkinson’s disease (PD) can be as disabling for an individual as their motor symptoms, if not more so [1]. Indeed, non-motor symptoms dominate the clinical picture of PD and contribute to severe disability, impaired quality of life, and shortened life expectancy [2]. Anxiety and depression are the most prevalent non-motor symptoms in PD. Depending on the criteria used, depression affects up to 50% of people affected by PD [3] and up to 31% of people with PD report some level of anxiety [4]. Even when people do not experience significant psychological disorders, they may still struggle to adjust to the social, emotional, and personal changes brought on by the condition.

Pharmacological interventions for anxiety and depression have received the most empirical attention to date. However, people with PD benefit less from antidepressant treatment than do patients without PD [5]. Also, there is a high risk of negative side effects and adverse interactions between antidepressants and antiparkinsonian medications [6]. Anxiety is managed pharmacologically largely also by antidepressants [7]. Atomoxetine, an effective drug treatment for anxiety in non-PD patients, was not found to be efficacious for anxiety in PD [8]. Benzodiazepines, commonly used for anxiety disorder treatment, are not recommended for PD patients due to adverse effects including cognitive and psychomotor impairment [9]. Therefore, non-pharmacological interventions can provide a good alternative for mood problems in PD.

To date, there is very little research into the effectiveness of psychological interventions in PD, with only a small number of studies [10–12] investigating the feasibility and effectiveness of cognitive behavioural therapy (CBT) in the treatment of depression and anxiety. Mindfulness-based group therapy is a rapidly growing psychological approach to helping people adjust to chronic illness and manage unpleasant symptoms that is embedded within the concept of acceptance. Mindfulness focuses on finding a new way to relate to thoughts and to accept them as passing events that do not necessarily represent a state of reality [13]. Mindfulness is based on the philosophy that human suffering develops in part by efforts to struggle with and avoid our own psychological and emotional pain. Changing one’s relationship to thoughts appears to be the most effective component of mindfulness-based intervention [13]. Over a typical 8-week mindfulness course, participants complete daily mindfulness meditation practices and attend weekly group meetings.

Furthermore, mindfulness has potential cost advantages as interventions are delivered to groups requiring less therapist time per patient. Mindfulness courses have clear time boundaries, and once the techniques have been taught, patients can continue practising without additional contribution from a therapist.

A 2005 meta-analysis of the health benefits of mindfulness across a range of chronic illnesses suggested that mindfulness is at least moderately effective at improving symptom management, anxiety, and depression in a range of conditions [14]. Three recent small randomised control trials (*n* = 29, 30, and 14) showed a significant decrease after the mindfulness-based intervention in reported motor symptoms [15, 16] and symptoms of depression [16, 17] and anxiety [16] for people with PD. A qualitative study evaluating the acceptability and feasibility of this approach in a group of patients with PD [18] has found that this form of intervention is well accepted by patients with PD. Further, an MRI study showed that mindfulness in PD leads to structural brain changes [19]. Increased grey matter density was found in the mindfulness group compared to the usual-care group in the right amygdala and bilaterally in the hippocampus [19]. These areas have been postulated to play an important role in PD as they seem to contribute to clinical motor symptoms (bradykinesia, tremor, rigidity) and non-motor symptoms.

To date, there is very little research in evaluating the effects of mindfulness in reducing anxiety and depression in patients with PD. Given the body of evidence exposed above, it would be realistic to expect that a mindfulness-based intervention could be beneficial for people with PD in relation to issues such as denial and acceptance of the illness, the loss of independence, and the many disturbances that go together with a disease over which the person has limited control.

We propose to deliver a mindfulness intervention via Skype, an online application that enables videoconferences with two or more people. Online mindfulness courses might facilitate attendance and adherence in this particular population [17]. There has been increasing use of distance-delivered interventions for people with disease-related barriers such as impaired mobility. Further, there is evidence that distance-delivered mindfulness interventions can also be effective in people with chronic medical conditions [20–23].

We ran two patient advisory group meetings (*n* = 14) with people with PD in preparation for this trial. Participants of these meetings were recruited through Parkinson’s UK research network and most had some knowledge of mindfulness and its application. The first and second authors facilitated the groups. We talked to participants about our plans with the trial and the development of the intervention, and here, we incorporated their feedback. The focus group participants talked about mindfulness being beneficial to people who do not necessarily experience high levels of anxiety or depression but still they have difficulties adjusting to PD challenges and managing PD symptoms. Participants also reported that although using Skype may exclude people not familiar with technology or people who do not own a computer, this method of delivery would make it possible for people across the country and those with restricted mobility to participate. Patient advisory group participants were overall in favour of using Skype and as one participant said ‘we need to move with the times, we need to go with the technology’.

### Aims

The overall objective of this study is to assess the feasibility of procedures and processes as well as potential efficacy (i.e. explore potential for detecting change in each of the proposed measures for each group) of a mindfulness-based intervention (MBI) for people with PD, which will be delivered via Skype.

The specific aims of this trial are to:

1. Monitor rates of recruitment, retention, refusal, adherence to the mindfulness sessions, and homework practice, as well as the appropriateness of eligibility criteria to determine the rate of each for planning the main trial
2. Assessing time and resource problems, for example whether it is feasible for patients to answer all the questionnaires and the time taken to do so, the flow of participants entering the study, the use of equipment to set up Skype, and other resources needed
3. Determine data management issues, by monitoring levels of missing data and data variability
4. Evaluate the potential for detecting a change in each of the proposed outcome measures (anxiety, depression, pain, fatigue, insomnia, quality of life, subjective well-being, physical impact of PD, health costs) when comparing the mindfulness-based intervention (MBI) group to the wait-list control (WLC) group
5. Understand the mechanisms of change (mindfulness skills, acceptance, self-compassion, decentering, tolerance of uncertainty) in each patient group, including any potential nocebo effects in the WLC group
6. Assess the qualitative experiences of receiving the mindfulness intervention and people’s views and experiences of using Skype as a mode of delivery as well as people’s feedback on the questionnaires used

## Methods/Design

### Design and theoretical framework

**Fig. 1:**
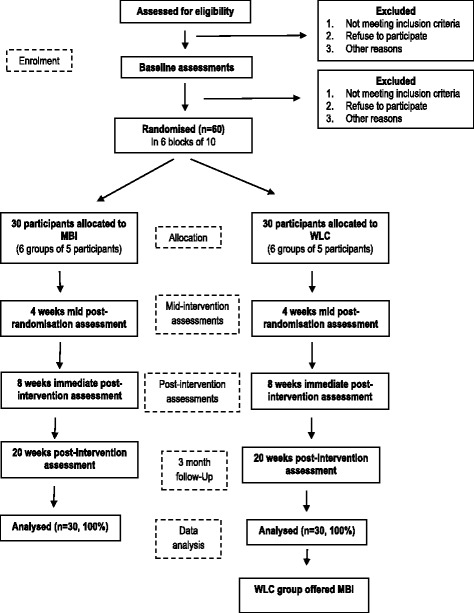
CONSORT flowchart of trial design

We will use a parallel group randomised controlled design. A total of 60 participants with PD will be randomly assigned to either an 8-week MBI course (*n* = 30) or a WLC (*n* = 30). CONSORT flow chart of the study is summarised in Fig. 1.

### Participants

#### Study sample

A pilot phase II trial of at least 30 participants is considered adequate for obtaining reasonably reliable sample size estimates [24]. We aim to recruit 60 people affected by PD. This sample will allow us to include six intervention groups to estimate intraclass correlations that relate to the fact that we are treating people in groups rather than individually.

#### Inclusion criteria

Participants included in this study must:

- Be diagnosed with PD according to UK PD Brain Bank Criteria [25, 26] by a neurologist or geriatrician
- Have a computer and internet access at home
- Be able to communicate in English fluently
- Be stabilised on PD medication, antidepressants, or anxiolytics (if taken) indicated by a stable dose for a minimum of 1 month

#### Exclusion criteria

Participants will be excluded from the study if they:

- Have severe cognitive impairment that would make participation in the mindfulness sessions and home practice of mindful meditation problematic or distressing. This will be assessed using the Telephone Interview for Cognitive Status Instrument modified version (TICS-M, [27]). People with a score of less than 20 will be excluded.
- Have serious psychiatric conditions (e.g. psychosis, drug/alcohol abuse) that can potentially risk failure in the intervention or limit participation in the course.
- Have severe hearing impairment.
- Are currently participating in other psychological therapies.
- Have prior formal training in mindfulness methods or current meditation practice.

#### Recruitment and consent process

Potential participants affected by PD are recruited through adverts on the Parkinson’s UK website and the Michael J. Fox Foundation. Recruitment started in February 2016 and is expected to finish in June 2016.

People with PD who see the web advert or the email during the trial period and are interested in the study will get in touch with the trial coordinator. Participants will receive adequate information before consenting to take part in the study, including information sheets and discussion with the trial coordinator. Potential participants will be issued with a Participant Information Sheet and will be encouraged to read it carefully and to discuss it with others before making a decision. Participants will also be encouraged to contact the researchers with any questions that they might have. In the initial discussion with the participant, the trial coordinator will check whether the participant has understood the purpose and nature of the research and what the research involves, its benefits (and lack of benefits), risks, and burdens. After the initial discussion with the trial coordinator and consideration of the information sheets, potential participants will have at least 24 h to fully consider the implications of taking part in the research, ask questions, and reflect, before giving consent. Individuals participating in the study will sign a written consent form before the baseline assessments. On the form, participants are given the choice to consent to the mindfulness trial and questionnaire assessments only or to an additional in-depth telephone interview after their mindfulness programme has been completed.

#### Eligibility screening and enrolment

During the initial telephone contact, the trial coordinator will explain the purpose of the study in detail and potential participant’s questions will be answered. After obtaining a verbal consent and explaining what will happen with the data given in the case of eligibility/ineligibility, the trial coordinator will administer the screening questionnaires via telephone. After screening, participants will be notified whether they are eligible or ineligible for the trial. If the results of the screening assessment indicate that they are eligible for the study, they will be given an invitation to participate and the trial coordinator will check whether they need any help with setting up Skype and clarify technicalities for the sessions.

Participants will be sent the equipment they need to use Skype, such as headsets and web-cameras (if not incorporated in their computers) as well as a booklet with instructions on how to set up Skype on their computer.

#### Randomisation

Participants will be randomly assigned to either the MBI group or the WLC group with a 1:1 allocation using a computer-generated blocked randomisation scheme. The randomisation scheme will be generated using the randomization.com website http://www.randomization.com. Block randomisation will allow keeping groups in similar sizes, ensuring a balance of numbers in each group at any time during the trial. Six blocks of 10 participants each will be used. The parameters will be saved for our records.

#### Booking in participants’ groups

On receiving the randomisation outcome from the research trial coordinator, the first author will contact the participants to notify them of their group allocation. Participants in the MBI group will be offered a date for their first session. Participants in the WLC group will be discouraged from any new mindfulness-related activities during the trial.

### Mindfulness-based intervention for PD

The MBI will be delivered in eight sessions over an 8-week period. The sessions will last 1 h and will be held via videoconference through Skype, an online application, in groups of five people. The videoconferences will be initiated by AB and will be arranged at a day and time that is most suitable for all participants in the group. CDs with homework mindfulness practices will address potential issues people with PD may come across during practices, like resting tremor, spasms, fatigue, and mind wandering.

The sessions will be carried out based on a written manual. However, flexibility will be allowed to follow issues and topics that are more important for the group during the sessions. Participants will be issued the mindfulness manual before their first session and will be encouraged to read only the chapter of the relevant session each week. The manual will contain eight chapters. In each chapter, the homework for each week will be outlined followed by a brief description of the week’s theme.

Participants will sign a consent form indicating that they understand that they are being recorded and that recordings will be stored in password-protected files and only be used for the stated purposes (i.e. fidelity checks and supervision).

#### Developing of the MBI manual and meditation CDs—new adaptations

The MBI manual is based on the mindfulness-based cognitive therapy (MBCT) programme for depression developed by Segal et al. [13]. In this study, we are using this MBCT programme but tailored to the needs of people with PD. One of the main changes we have introduced is the reduction of time in the mindfulness home practice. The original MBCT programme suggests that participants should practise mindfulness meditation for 45 min, 6 days a week. Due to their condition, people with PD may have difficulties maintaining a long practice, due to problems with sitting still in one posture, maintaining concentration, and fatigue. Therefore, the meditation practices were shortened to 20 min to accommodate the needs of people with PD. Further, we have reduced the weekly group sessions from 2/2.5 to 1 h. It has been found that the correlation between mean effect size and the number of in-class hours is non-significant for both clinical and nonclinical samples, which suggests that adaptations that include less class time may be worthwhile for populations for whom longer time commitment may be a barrier to their ability to participate [28]. In fact, shortened class time has been used successfully before, for example in people with multiple sclerosis [23], multiple sclerosis and peripheral neuropathy [28], and PD [17]. The manual also includes PD-specific examples. These changes will make the intervention more accessible for people with PD without compromising its effectiveness.

#### MBI sessions

AB will deliver the 8-week course. AB is a health psychologist, who has completed teachers’ training to deliver mindfulness-based courses and has experience delivering mindfulness programmes for people with neurological conditions via Skype [23]. LM, a clinical psychologist with experience in mindfulness programmes for people with long-term conditions, will provide supervision.

This MBI course consists of eight sessions that cover the following topics: (1) automatic pilot, (2) mindfulness of breath, (3) staying present, (4) dealing with barriers, (5) using mindfulness to respond instead of react, (6) thoughts are not facts, (7) how can I best take care of myself, and (8) planning for the future. For more details on the content of the sessions, see Table 1. Recordings of three classic meditations will be provided including the body scan, a sitting meditation, and a mindfulness movement meditation for daily home practice.

**Table 1:** Summary of the content of the MBI manual for P

Week	Theme	Agenda
Week 1: Automatic pilot	Mindfulness starts when we recognise the tendency to be on automatic pilot and make a commitment to learning how best to step out of it to become aware of each moment. Body scan practice trains the mind in putting attention and awareness in different places at will. The aim of this week is to enhance awareness.	Establish the orientation of the group • Set ground rules regarding confidentiality and privacy • Ask participants to introduce themselves in the group • Raising exercise • Discussion of raising exercise • Body scan meditation Homework: body scan meditation (6 out of 7 days), mindfulness of a routine activity, eat one meal mindfully.
Week 2: Mindfulness of the breath	Further focus on the body begins to show more clearly the chatter of the mind and how it tends to control our reaction to everyday events. The aim of this week is to enhance awareness.	• Body scan meditation • Homework review • Cultivating curiosity about body sensations • Hearing meditation Homework: body scan (6 out of 7 days), pleasant events calendar, and mindfulness of a routine activity.
Week 3: Staying present	Greater awareness of how the mind can get hooked in judgements and unhelpful thoughts leads to awareness of the impact of those thoughts and emotions on the body. In this week, decentering is introduced.	• Mindfulness movement meditation • Homework review • Explain low mood/anxiety cycle and the role of automatic thoughts • 3-min breathing space Homework: body scan or mindful movement meditation for 6 days out of 7, unpleasant events calendar, 3-min breathing space.
Week 4: Dealing with barriers	The mind is most scattered when it tries to cling to some things and avoid/escape other things. Mindfulness offers a way of staying present by giving another place from which to view things: to help take a wider perspective and relate differently to experience. In this week, decentering is further explored and acceptance is introduced.	• Sitting meditation • Homework review • Explain acceptance versus avoidance/fusion concepts and explore unhelpful coping strategies linked to avoidance or fusion. • 3-min breathing space-coping Homework: sitting meditation (6 out of 7 days), 3-min breathing space, early warning signs and action plan exercise.
Week 5: Using mindfulness to respond instead of react	Relating differently involves bringing to experience a sense of ‘allowing’ it to be, just as it is, without judging it or trying to make it different. Such an attitude of acceptance is a major part of taking care of oneself and seeing more clearly what, if anything, needs to change. Acceptance is further explored, and self-compassion is introduced.	• Sitting meditation • Homework review • Responding vs. reacting • 3-min breathing space-dealing with difficulties Homework: sitting meditation (6 out of 7 days), 3-min breathing space.
Week 6: Thoughts are not facts	Negative moods and the thoughts that accompany them restrict our ability to relate differently to experience. It is liberating to realise that our thoughts are merely thoughts, even the ones that say they are not. This week brings together the concepts of decentering, acceptance, and self-compassion.	• Sitting meditation • Homework review • Thoughts and feeling exercise (‘walking down the street’) • Discuss how decentering and accepting can lead to being kinder towards oneself • 3-min breathing space Homework: practise with a selection of meditations, 3-min breathing space.
Week 7: How can I best take care of myself?	There are some specific things that can be done when distressed. Taking a breathing space will come first and then deciding what action, if any, to take. Each person has his or her unique warning signs of distress, but participants can help each other in making plans of how best to respond to the signs. This week brings together the concepts of decentering, acceptance and self-compassion.	• Sitting meditation • Homework review • Generate list of pleasure and mastery activities • Plan how best to schedule such activities • 3-min breathing space as the first step before choosing whether to take mindful action Homework: Select from all different forms of practice, a pattern you intend to use on a regular basis, 3-min breathing space.
Week 8: Planning for the future	Maintaining a balance in life is helped by regular mindfulness practice. Good intentions can be strengthened by linking such intentions to a positive reason for taking care of oneself	• Body scan meditation • Homework review • Review whole course: what has been learned • Discuss how best to keep up momentum and discipline developed over the past 7 weeks in both formal and informal practice • Concluding meditation

The sessions themselves will be based on discussion of participants’ experiences with their meditation practice and mindfulness concepts. Each session will start with a short meditation practice, followed by a brief discussion about the practice just completed and the homework practice of the previous week. Then new concepts will be introduced, such as acceptance, relating to thoughts, and self-compassion. Another short meditation practice will follow this discussion, and at the end, homework for next week will be set.

#### Missed sessions and homework

AB will record attendance and make notes on participation. Details of issues covered and homework engagement will be recorded as well. In the case of non-attendance at sessions, AB will contact the participant to ascertain the problem and to discuss a suitable solution addressing any concerns. Unfortunately, because of the group setting of this intervention, participants will not have the opportunity to reschedule a missed session.

Participants will be asked to keep a diary (provided with the manual) of their weekly homework mindfulness practices. A record of homework engagement will be kept, and final analyses will control for homework compliance.

### Wait-list control group

9pt?>Participants allocated to the WLC group will receive the treatment they would normally expect from the National Health Service (NHS) in the UK. People may receive a mix of clinical input and review from both primary and secondary care providers, according to individual health needs. Access to psychological assessment and intervention for people with PD is inconsistent across the UK, and dedicated psychological services are extremely scarce [29]. People in the WLC group will be offered the opportunity to take part in the MBI at the end of the 20-week follow-up.

### Assessments

DVS, the trial coordinator, not involved in the therapy sessions, will coordinate all the assessment procedures. As shown in Table 2. Participants will be asked to complete a set of standardised questionnaires online or on paper, depending on their preference, at four time points (baseline, mid- and end of the intervention, and 3 months after completing the intervention). If participants are unable to use a keyboard or write on paper, they will have the option of completing assessments by telephone.

#### Screening measure

Telephone Interview for Cognitive Status Instrument modified version (TICS-M, [26]). This 13-item instrument will be used to assess cognitive status over the phone. Although this screening tool gives particular emphasis on learning and memory, items probing orientation, repetition, naming, and calculation are also included. The total score ranges from 0 to 50, with higher scores indicating better cognition. A total score cut-off of 20 will be used to deem participants eligible to take part in the study.

#### Feasibility outcomes

Feasibility outcome measures have been decided following Thabane et al. [30] recommendations.

We will compare the actual recruitment rate to the target recruitment rate, which is 10 participants per month, and also compare the actual recruitment rate to recruitment rates of psychological trials in PD. We will also compare the retention/refusal and adherence rates to other psychological trials in PD as well as mindfulness interventions in PD delivered face-to-face. We will consider the trial feasible if these processes compare favourably to other psychological trials or mindfulness trials in PD. We will also assess the appropriateness of the eligibility criteria in recruiting a population receptive to, and appropriate for, a mindfulness intervention by assessing adherence and retention rates. We will assess whether the criteria were too restrictive by comparing recruitment targets against actual recruitment process and also assessing whether too many people were excluded based on specific criteria.

We will assess time and resource problems that can occur during the main study by assessing the length of time required to fill out all the questionnaires. We will investigate whether there is participant overload, how much time it takes to screen participants and mail out consent forms, whether Skype equipment is needed, and how problems with installing or using Skype can best be managed.

We will also investigate issues around data collection, whether data from different data points can be matched, whether diary entries can be logged accurately, whether there are important data variables left blank, or whether the data show too much or too little variability.

#### Patient-centred outcome measures

We will explore the potential for detecting change in each of the following outcome measures for each group separately.

##### Hospital Anxiety and Depression Scale (HADS, [31])

This 14-item scale will be used to assess symptoms of depression (7 items) and anxiety (7 items). This scale has been found to be effective in assessing the symptoms of the severity of anxiety and depression in both secondary and primary care patients [32]. Each item is scored on a scale of 0–3, with 3 indicating higher symptom frequencies. Scores for each subscale (anxiety and depression) range from 0 to 21. A cut-off score of at least 8 for each subscale will be used as an indication of caseness of depression and/or anxiety. HADS is likely to be the primary outcome in the main trial.

##### Brief Pain Inventory (BPI, [33])

Pain severity/intensity as well as pain interference with daily life will be assessed with this numerical rating scale (scaled from 0 to 10). This scale assesses the average pain, which is associated with PD according to the patient’s point of view. Thereby, 0 represents no pain and 10 the most painful sensation imaginable.

##### Fatigue Severity Scale (FSS, [34])

This nine-item scale will be used to assess the impact of fatigue on the daily living of patients. It comprises three items related to physical impact, three items to the psychological environment, and the remaining three are more generic. Each item is rated on a 7-point Likert scale (1 = completely disagree–7 = completely agree). Mean scores of 4 or more define significant fatigue.

##### Insomnia Severity Index (ISI, [35])

This self-report measure consists of seven items that assess the nature, severity, and impact of insomnia in the last 2 weeks. Each item is rated on a 5-point Likert scale (0 = not at all–4 = extremely) with total scores ranging from 0 to 28, whereby higher scores indicate greater insomnia severity.

##### Parkinson’s Disease Activities of Daily Living Scale (PADLS, [36])

This self-report scale will assess difficulties in daily activities due to PD. This five-item scale provides a single global rating of how patients perceive their illness, with higher scores indicating greater difficulty in activities of daily living.

#### Process mechanism measures

We will also assess some mindfulness-related mechanisms. Participants will be asked to keep a diary in which they will monitor the frequency and duration of their home mindfulness practice.

##### Self-Compassion Scale—Short Form (SCS-SF, [37])

Research has shown that self-compassion is associated with psychological well-being and suggests that self-compassion might be an important protective factor, fostering emotional resilience (see Neff, [39], for a review). Self-compassion will be assessed using 12 items and a total score will be generated. This short form of the SCS demonstrates adequate internal consistency (Cronbach’s alpha ≥ 0.86) and a near-perfect correlation with the long form SCS (*r* ≥ 0.97) [37].

##### Experiences Questionnaire (EQ, [40])

This questionnaire measures decentering, one of the process mechanisms that are used during the MBI course with the view to reduce distress. Decentering is defined as the ability to observe one’s thoughts and feelings as temporary, objective events in the mind, as opposed to reflections of the self that are necessarily true. An initial validation study of EQ showed that levels of decentering among people with major depression were significantly and negatively correlated with concurrent self-report (*r* = −0.46) and clinician-assessed (*r* = −0.31) levels of depression symptoms.

##### Acceptance Action Questionnaire (AAQ-II, [41])

The AAQ-II assesses acceptance, experiential avoidance, and psychological inflexibility, which is related to a range of outcome, from mental health to work absence rates. Results from 2816 participants across six samples indicate the satisfactory structure, reliability, and validity of this measure [42].

##### Philadelphia Mindfulness Scale (PHLMS, [43])

This 20-item scale comprises two subscales: awareness and acceptance. The awareness subscale assesses noticing and being aware of thoughts, felons, perceptions, and body sensations, while the acceptance subscale assesses experiential avoidance.

##### Intolerance of Uncertainty Scale—Short Version (IUS-12, [44])

The 12-item scale measures the participant’s response to uncertainty, ambiguous situations, and the future. It is rated on a 5-point Likert scale ranging from 1 (not at all characteristic of me) to 5 (entirely characteristic of me).

##### Home practice diary

Participants will be asked to keep a diary (either on paper or online) in which they will monitor the frequency and the duration of their home mindfulness practice.

#### Health economics measures

Participants of both arms will be measured using the assessments described below.

##### Subjective well-being (SWB, [45])

These four items from the Office of National Integrated Household Survey, 2011, will assess feelings regarding participants’ life satisfaction, happiness, anxiety, and life as something worthwhile at baseline, pre-, and post-intervention time points.

##### EuroQol five-dimensional questionnaire and visual analog scale (EQ-5D-3L and EQ-VAS, [46, 47])

The EQ-5D-3L is a five-item composite measure, and the EQ-VAS is a visual analog scale, both developed by the EuroQol Group. The EQ-5D-3L consists of five dimensions (mobility, self-care, usual activities, pain/discomfort, and anxiety/depression) each with three levels of problems. Both the EQ-5D-3L and EQ-VAS will be used to assess participants’ quality of life.

##### The ICEpop CAPability measure for Adults (ICECAP-A, [48]) and the Adult Social Care Outcomes Toolkit (ASCOT, [49])

The ICECAP-A is a measure of capability for the general adult population (+18) comprised of five attributes of well-being (attachment, stability, achievement, enjoyment, and autonomy); the ASCOT is a self-compliance questionnaire comprised of eight attributes (personal cleanliness and comfort, food and drink, control over daily life, personal safety, accommodation cleanliness and comfort, social participation and involvement, occupation, and dignity).

#### Feedback interview procedure

At the end of the 8-week intervention period, participants, in the MBI group, will also be included to be interviewed by an MSc health psychology student who has not had any involvement in other aspects of the trial. Participants will be asked to give open and honest accounts of their experiences and opinions of the intervention (see topic guide, Table 3). Questions address expectations regarding the intervention, experiences of MBI sessions, features of the intervention liked and disliked, and the process of change (or not) as a result of the course. Each interview will be digitally recorded. Interviews will be anonymised and transcribed; any information that may compromise confidentiality will be omitted before the transcripts come to the research team.

**Table 2:** Details and schedule of the MindPD trial assessment procedure

Assessments administered	Screening (week 0)	Baseline (week 1)	Mid-intervention (week 4)	Post-intervention (week 8)	3-month follow-up (week 20)
Main details sheet	X				
Telephone Interview for Cognitive Status Instrument modified version (TICS-M)	X				
Demographics sheet		X			
Primary outcome measure:					
Hospital Anxiety and Depression Scale (HADS)		X	X	X	X
Secondary outcome measures:					
Parkinson’s Disease Activities of Daily Living Scale (PADLS)		X	X	X	X
Brief Pain Inventory (BPI)		X	X	X	X
Fatigue Severity Scale (FSS)		X	X	X	X
Insomnia Severity Index (ISI)		X	X	X	X
Process variables:					
Self-Compassion Scale (SCS)		X	X	X	X
Philadelphia Mindfulness Scale (PHLMS)		X	X	X	X
Experiences Questionnaire (EQ)		X	X	X	X
Acceptance Action Questionnaire (AAQ-II)		X	X	X	X
Intolerance of Uncertainty Scale (IUS-12)		X	X	X	X
Health economics variables:					
Subjective Well-Being questionnaire (SWB)		X		X	X
EuroQoL and visual analog scale (EQ-5D-3L and VAS)		X		X	X
ICEpop CAPability Measure for Adults (ICECAP-A)		X		X	X
Adult Social Care Outcomes Toolkit (ASCOT)		X		X	X
Home practice				X	X

### Data protection and confidentiality

Recordings, transcripts, and documentation relating to participants will be stored in a locked filing cabinet at PI’s office at City, University of London and only available to the research team. Each participant will be assigned a study ID that will be kept separate to a list with ID and names. Data stored on the computer will be password protected, and only members of the research team will know the password. In the write-up of the research, names and any other identifiable information will not be used.

### Blinding

The research trial coordinator will coordinate all assessment procedures. Data will be downloaded in an Excel spreadsheet by the trial coordinator. All assessments are based on self-report online assessments, rather than rated or imputing by a member of the research team. Therefore, influences of observer biases are not expected.

Due to the nature of the intervention, it is not possible to blind the principal investigator, as she will be delivering the MBI sessions. It is also not possible to blind participants to intervention allocation. During statistical analysis, the intervention groups will be assigned a code rather than their name (i.e. WLC vs. MBI) so that the researchers are unaware of what the intervention group code represents.

### Compliance

This pilot trial will be conducted in compliance with the Declaration of Helsinki, Medical Research Council Good Clinical Practice (GCP) guidance, the Data Protection Act (1998), the National Research Ethics Service (NRES) approvals, National Health Service (NHS) Trust regulations, and other regulatory requirements as appropriate. The final trial publication will include the items recommended under the CONSORT statement for randomised trials [50].

### Participant safety

This MBI course is expected to be of low risk to participants. Mindfulness approaches are non-invasive and have been previously used successfully in chronic illness populations [14]. If AB is concerned about a patient’s safety, a referral will be made to ensure the participant gets the support he/she needs.

#### Withdrawals from MBI course

If a participant expresses the wish to withdraw from the MBI course, AB will contact the participant to ascertain the reason for drop-out if the participant is willing to share this.

The reason for withdrawal (e.g. adverse events, illness progression, inability to adhere, inability to attend) will be discussed with all team members, and the information will be recorded as appropriate. Again, if it is felt that a participant should be withdrawn from the course, this will be discussed in a meeting with all the members of the research team.

### Data analysis plan

#### Quantitative analysis plan

Statistical analysis will be carried out in STATA. Descriptive statistics (mean, median, standard deviation) will be calculated for all demographics and clinical variables. All group comparisons will be carried out on an intention-to-treat basis, that is participants will be analysed in the group to which they were randomised. Descriptive results concerning recruitment numbers, completions, drop-out rates, and missing data will be calculated and reported. The quantitative data will be summarised with standard descriptive measures for each group separately to determine potential change within group over time. Continuous variables will be summarised by their mean and standard deviation, categorical variables as numbers and percentages, and presented with 95% confidence intervals. With the aim of identifying a potential response shift in some of the participants, a retrospective measurement will also be implemented. In particular, then-test questions will be added to the post-intervention and 20-week follow-up questionnaires for several outcome variables. Both measurements of the effect of the course (prospective and retrospective) will be compared, in order to add robustness to the results. We will also assess the sensitivity of the results to excluding patients who did not receive a sufficient number of MBI sessions or those who had little meditation home practice.

**Table 3:** Topic guide for participants’ experiences with the mindfulness course

Questions	Prompts
1. First of all, can you start by telling me what you were expecting from the mindfulness sessions?	-What did you think the programme would be like? -In what ways (if any) did you think it might help you?
2. How did you find the programme overall?	-Tell me how you found your first session -Tell me about the other sessions -Tell me how you found the homework tasks
3. Can you tell me what you liked about the programme?	-What was helpful? Why? How? -Were there some sessions/some aspects that were more helpful than others?
4. Can you tell me what you disliked about the programme?	-What was unhelpful? Why? How? -Were there some sessions/some aspects that were less helpful than others?
5. Tell me about anything that you feel has changed from having done the programme?	-Can you tell me what changed? (Anything different in your day-to-day life, the way you are dealing with PD?) -Can you tell me how you came to notice things changing? -Why/how do you think things changed?
6. Do you have anything else you would like to tell me about your experiences of this programme that have not already covered?	-What would you feed back to the people who put together the programme? -What advice would you give to people thinking about taking part in mindfulness-based programmes?
7. What do you think of the questionnaires used and the overall set-up of the study?	-How did you find participating in a course over Skype? -Any further comments regarding the questionnaires used?

#### Economic evaluation plan

Cost comparisons will be made between the mindfulness course and the standard care. Service cost data will be combined with the primary outcome measures (HADS) and quality-of-life measures generated from the EQ-5D-3L to assess cost-effectiveness. If the intervention has lower costs and better outcomes, then it will be ‘dominant’. In the event of the intervention having higher service costs and better outcomes, cost-effectiveness will be assessed using incremental cost-effectiveness ratios. To address uncertainty in cost and outcome differences, we will run different sensitivity analyses, as tornado diagrams, worst-best scenarios or cost-effectiveness acceptability curves. The results of this economic evaluation should be treated with caution as the study is underpowered. However, the results will give an indication of the potential cost-effectiveness of the mindfulness intervention.

#### Qualitative analysis plan

The interviews will be analysed using inductive thematic analysis. The inductive analysis is a process of coding the data without trying to fit it into a pre-existing coding frame or the researcher’s analytic preconceptions [51]. The analysis will be conducted following Braun and Clarke’s guidelines [52]. The analysis of the transcripts will be conducted in parallel with ongoing data collection. First, each coding unit in the first transcript will be given a code name, using vocabulary as close as possible to that used by participants themselves [53] to avoid prematurely importing preconceptions into the analysis. This procedure will be repeated on the second transcript. When the same themes reoccur, they will be provided with the same label. Initial codes will then be applied systematically to the entire dataset, giving full and equal attention to each data item. As data analysis, proceeds codes will be re-defined as new and alternative themes arise. Earlier transcripts will be re-coded as codes are developed and refined. During this analysis, the validity of individual themes in relation to the dataset will be considered and also whether the themes reflect the dataset as a whole. A detailed paper trail will record the development of the codes and the relationship between the raw data and the refined themes and codes. Further, framework analysis techniques [54] will be used, where the final codes will be tabulated to inspect the data for patterns and relationships in the themes between people with PD.

## Discussion

Mindfulness-based interventions have been shown to effectively reduce anxiety, depression, and pain in patients with chronic physical illnesses, including PD [14]. Mindfulness-based interventions have been traditionally delivered in a face-to-face group setting [55]. This pilot trial will look specifically at assisting psychological adjustment for people with PD and is conducted in preparation of an efficacy study for an online MBI in PD. The distance delivery of this trial means that we are offering psychological support to a group of patients who would not otherwise routinely have formal access to psychological interventions but who nonetheless are faced with considerable psychological challenges. With this study, we will explore different ways to deliver group psychological interventions and we will enrich our knowledge on the specific needs and challenges faced by people affected by PD as well as how best to address these needs.

Some limitations of the current study need to be acknowledged. First, we will not have a large enough sample to account for the heterogeneity in the non-motor challenges that people with PD face. Therefore, we will not be able to make sub-group comparisons of the potential effectiveness of the interventions for people who entered the trial with different non-motor challenges. Further, a WLC group can induce nocebo effects [56], inflating the effectiveness of the intervention. Any worsening of symptoms for the WLC group will be reported and suggestions will be made for more appropriate controls in a future larger trial. Also, the total HADS [31] score may not be the most appropriate tool to detect caseness of depression and anxiety [57]. However, it was chosen since HADS measures anxiety and depression without confounding by somatic symptoms of the physical disorder, in contrast to other depression and anxiety scales that include items like insomnia, retardation of psychomotor, tremor, dry mouth, muscle aches, loss of energy, and fatigue. These items although they are an indication of anxiety or depression, they can also be symptoms of PD and not a reflection of anxiety or depression. There are also some validation studies for HADS showing good psychometric properties [58–61]. In addition, HADS will potentially be chosen as the primary outcome of the intervention and it was important to choose a measure that is sensitive to change both during the course of an illness and in response to medical or psychological intervention [33, 62]. HADS is also relatively short and easy to administer. Finally, Skype calls depend on the strength of the internet connection and can interrupt therapy sessions. AB has experience delivering these courses over Skype without excessive technical problems [23, 63]. AB usually incorporates any disruptions or lack of quality as examples that participants could use to practise staying with difficulties and identify thoughts arising when there is frustration, so these interruptions become part of the mindfulness learnings. The qualitative interviews with participants at the end of the courses will help us explore experiences of the course and the Skype delivery. These interviews will direct us to potential changes in the future courses.

Nevertheless, this pilot trial will answer important questions about the feasibility of procedures and processes as well as the potential efficacy of an 8-week distance-delivered mindfulness course in reducing distress and increasing the quality of life. If the results show that this intervention is feasible and potential effective for people with PD, a grant will be prepared for a large randomised control trial which will aim to assess the effectiveness of this intervention on a representative sample and compared against an active control group. We will also explore the role of different ‘active ingredients’ of mindfulness-based approaches such as acceptance, self-compassion, decentering, and cultivating mindfulness skills to determine how mindfulness interventions work and which are the most important components of these interventions regarding changes in outcome measure. Further, this pilot trial will provide some indication regarding the cost-effectiveness of the intervention and its acceptability to patients.

This pilot trial is set up to be methodologically robust and to conform to CONSORT guidelines. We have taken care to address key sources of bias; the MBI for PD course will be subject to ongoing supervision to ensure therapy fidelity, and this will also be checked post-therapy by independent raters. The assessments of patient outcomes are self-reported and collected by an independent researcher who is uninvolved in therapy delivery. The MBI trial for PD also benefits from service-user input from the early stages of therapy design and manual development, right through to the in-depth evaluation of the MBI sessions. The data from the feedback interviews will be particularly important in guiding any changes to the MBI format.

## Abbreviations

CBT: Cognitive behavioural therapy
GCP: Good Clinical Practice
MBI: Mindfulness-based intervention
MBCT: Mindfulness-based cognitive therapy
NHS: National Health Service
NRES: National Research Ethics Service
PD: Parkinson’s disease
WLC: Wait-list control
